# The role of environment on women’s perception about their STEM studies: observations from a Global South country

**DOI:** 10.1038/s41598-023-50571-w

**Published:** 2024-01-02

**Authors:** Mariza Tsakalerou, Asma Perveen, Alibek Ayapbergenov, Aida Rysbekova

**Affiliations:** 1 School of Engineering and Digital Sciences, Nazarbayev University, Astana, Kazakhstan; 2 School of Engineering and Digital Sciences, Nazarbayev University, Astana, Kazakhstan; 3 Nazarbayev University, Astana, Kazakhstan

**Keywords:** Psychology, Human behaviour

## Abstract

The growing global demand for STEM professionals is not being met by the supply of new graduates, a supply that is characterised by a significant lag in the percentage of women pursuing STEM studies. Interestingly enough, the percentage of female applicants entering STEM majors has been increasing yet only a minority of them pursue, or complete, engineering programs. Several studies for the developed world have identified several environmental factors responsible for this phenomenon. The scarcity of engineering professionals is a handicapping factor for development, even for the most advanced countries of the Global South. The objective of this exploratory study is to examine whether the environmental factors identified in the international literature are sufficient to explain the asymmetry in selecting an engineering or a natural sciences career among female undergraduates in an exemplary Global South country, Kazakhstan. To this purpose, a multifaceted survey was conducted among the female students pursuing STEM majors in the premier Kazakhstani university in the academic year 2021–2022. This study utilized a Likert Scale questionnaire, ordinal logistic regression, and factor analysis to explore factors affecting female students. Data reliability was confirmed through Confirmatory Factor Analysis (CFA). The factor and regression analysis of the results obtained demonstrates that there is no discernible difference between the observations in the literature and the situation in Kazakhstan.

## Introduction

In recent years, the topic of gender equality and women’s empowerment has been on the agenda of numerous conferences and fora. This issue is also included in the list of sustainable development goals initiated by the United Nations (UN) as a standalone goal aimed at eliminating all forms of discrimination against all women and girls everywhere. Gender parity is lacking across various organizational structures, including the field of education^1^. This educational polarization is particularly evident in the number of female and male students pursuing Science, Technology, Engineering, and Mathematics (STEM) disciplines globally. Within the global enrolment of female students in tertiary education, only around 30% account for the STEM disciplines^2^.

The issue is critically important for countries that belong to the Global South, that is the list of countries whose economies are not yet fully developed, and which face specific socioeconomic challenges^3^. The Global South concept is relevant to Central Asia in multiple ways. In fact, recent research has shifted the focus from correctly classifying individual Central Asian countries to addressing the many facets of the region’s “southernness”^4^ most notably its lagging innovation performance. Interestingly, innovation performance has emerged as a key factor distinguishing the Global South from the Global North^5^.

Kazakhstan, the largest country in Central Asia, has shown remarkable economic performance since its independence in 1991, transitioning from a lower-middle-income country to an upper-middle-income country. This development trajectory was primarily based on the country’s abundant hydrocarbon reserves and mineral resources^6^. To mitigate the heavy reliance on natural resources and to keep up with the global innovation pace, radical shifts are required in the educational sector to prioritize STEM disciplines at high schools and universities. The lower presence of women in engineering and manufacturing within the broad spectrum of STEM fields results in an absence of diversity and inclusion in workplaces^7^. The resulting outcome is a suboptimal performance in innovation creation within the country^8,9^.

**Figure 1:**
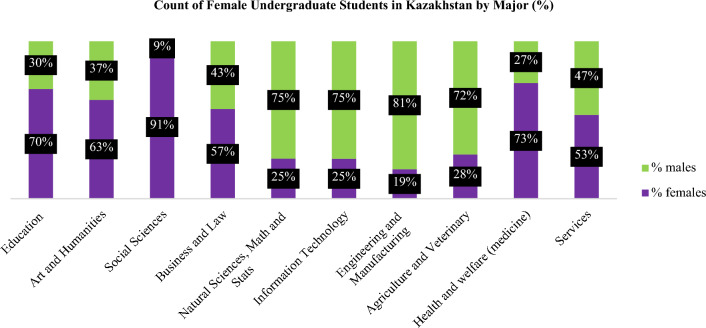
Proportion of undergraduate students by major in Kazakhstan in the academic year of 2022–2023.

Kazakhstan has implemented several policies and strategies as part of its efforts to achieve gender parity in various domains, including the establishment of a national organization that advocates for gender equality^10^. Figure 1 depicts the distribution of female students across different undergraduate specializations in Kazakhstan.

The findings depicted in Fig. 1 reveal a considerable high enrolment rate of female students in higher education. Notably, the fields of education, arts and humanities, social sciences, and medicine exhibit a significant preponderance of female participation, ranging between 60 to 90%. Conversely, despite the persistently enforced government strategies, female undergraduates who pursue engineering and manufacturing, and information technology (IT) majors are still predominantly outnumbered by their male counterparts. This is evidenced by the fact that women constitute only 19% and 25% of those pursuing these study disciplines, respectively^11^.

The structure of this paper is as follows: firstly, a literature review explores prevalent environmental factors influencing female students globally. The subsequent section includes theoretical framework based on relevant literature and methodological instruments of this study. This is followed by offering a comprehensive analysis and interpretation of the obtained results through a well-informed and substantiated discussion, along with limitations and suggestions for future research. Finally, the conclusions of this paper and some implications of the key results are presented in a summary form.

## Literature review

Despite continued initiatives and active participation of the Kazakh government in promoting STEM education by granting scholarships that cover full tuition fees to students, just 43% (17.4 k) of 40.5 k annual STEM graduates in 2021 entered the STEM labour market. Similarly, just around 8% of 38.1 k STEM undergraduate graduates enrolled in related STEM Master's programmes^12^. This phenomenon is referred to as the "leaky pipeline," and it implies that students abandon STEM pursuits at various stages: when STEM high school graduates choose non-STEM majors, when STEM undergraduate students graduate and decide to pursue another discipline at the Master's level, and finally when STEM graduates enter non-STEM careers^13^. This problem is more prevalent among females than males, resulting in an underrepresentation of women in STEM disciplines at various levels of the pipeline^14–16^.

The issue of female students’ under-representation in engineering, manufacturing, and IT disciplines traces back to traditional and cultural sentiments in Kazakhstan. Kazakh society has experienced a set of traditional and ideological influences throughout its formation as an independent state. It is a mix of nomadic, Islamic, Soviet, and recently emerging Western cultures^17^. Therefore, the cultural background of society might shape educational and working experiences differently from that of other countries. This is particularly evident in Kazakhstan’s traditionally male-dominated industries like engineering, manufacturing, and information technology^18^.

Many women experience the glass ceiling phenomenon, which is when they are unfairly prevented from being considered for high-paying jobs or advancing in their careers. According to the Federal glass ceiling commission, it is “the unseen, yet unbeatable barrier that keeps minorities and women from rising to the upper rungs of the corporate ladder, regardless of their qualifications or achievements”^19^. A study conducted in Kazakhstan reported more and more females focusing on their careers in order to gain financial independence, which comes with postponement of family life for some. Despite prioritizing careers over their personal life and sometimes putting off starting a family, female is still often stereotyped as having to work harder to be heard, seen, and taken seriously in comparison to their male colleagues. Another study conducted in the mining sector reported on decreased participation of females over the years^20^. Long maternity leave policies in Kazakhstan, lasting up to three years, also contribute to the additional challenges and biases faced by women in the workforce, impacting their ability to advance in their careers^21^. It can be difficult for women in Kazakhstan to balance their roles as a caregiver and a leader, all while facing a lack of recognition, support, and an overwhelming workload^22^.

Unfortunately, for many of the Global South countries, there are not enough studies to demystify the gender gap issues and provide insights into female contributions to development. Recent research papers focused on the scarcity of such studies and issued a call for targeted research in Global South countries^23,24^. The current body of literature on Kazakhstan's STEM education largely lacks a comprehensive examination of the factors that influence female students' intent to pursue or abandon STEM-related majors.

This research partially adopts the social cognitive theory framework which posits that human choice is shaped by the complex interplay between cognitive processes, environmental factors, and behavioural patterns^25^. The interplay of various factors in reciprocal determinism defines the three dimensions that ultimately influence human decision-making, including the choice to leave the STEM trajectory.

The initial phase of our literature review process was initiated by identifying specific keywords of significance. We initiated our exploration with terms including "STEM," "Gender gap," "Education," and "Retention." Subsequently, our search scope was expanded to encompass supplementary keywords such as "Human behavior," "Gender biases," and "Gender discrimination." To ensure a comprehensive literature review, we leveraged multiple scholarly search platforms, including but not limited to Google Scholar, PubMed, ProQuest, Scopus, and Web of Science.

Drawing from a comprehensive literature search, the environmental factors that potentially shape students' decisions include family dynamics, societal and cultural factors, career-related considerations, the campus environment, and the absence of female role models and mentors.

A negative campus climate can detrimentally impact students' emotional well-being and commitment, underscoring the need for inclusive and supportive educational environments^13,26^. Supportive peer relationships emerge as indispensable for female students' pursuit and completion of STEM degrees, emphasizing the role of interpersonal connections in fostering academic success^27,28^. Similarly, learning facilities play a vital role, with extracurricular activities and STEM-focused environments fostering interest and commitment among students^29^. This includes a specialized study curriculum, the availability of STEM school clubs, and STEM Olympiads, which sparked competitive interest among students^30^. Female mentors and role models also play a significant role in shaping women’s perceptions and motivations^31,32^. They are pivotal in bolstering young women's confidence and aspirations within the STEM domain, fostering a sense of belonging and identity^33^.

Equally critical is the role of family support and encouragement, which can either nurture or hinder students' STEM aspirations^28,32^. Drawing from the findings of the local context, it has been emphasized that facets of Kazakh culture place considerable weight on the involvement of both immediate and extended family members in determining a child's prospective academic pursuit. Regrettably, results obtained from the interviews indicate that STEM disciplines are frequently not endorsed by the family^34^. Such decisions could be influenced by the societal and cultural stereotypes that are intrinsic to the local culture, portraying women as obligated to prioritize familial responsibilities, such as caring for children and maintaining the household, over pursuing higher education and career growth^13,35^. Consequently, these stereotypes compel young women to choose an academic path regardless of their inherent passions and interests^30^.

Career-related barriers further hinder progress, with discriminatory practices perpetuating gender disparities in STEM fields^29,36,37^. Despite burgeoning opportunities in STEM-related industries in Kazakhstan due to a rapid industrialization^38^, deeply ingrained stereotypes and gender-based discrimination persist, leading to lower participation and unequal compensation for women^18^. For instance, in Kazakhstan, the gender wage gap ranges from 59% in the housing and catering industries to 91% in education, in favour of their male counterparts^39^. These discriminatory actions have adverse effects on women's ability to sustain their careers in STEM fields.

Given the absence of significant research in this area, the objective of this study is to investigate whether the factors that have been observed internationally as influential for female students in STEM fields are similarly relevant in the local context. Also, while several research studies are exploring the gender gap in STEM fields broadly in Kazakhstan, much less attention is paid to exploring this phenomenon in a more granular way using quantitative approaches.

Thus, the purpose of this study is thus to gather empirical data from Kazakhstan and scrutinize the environmental factors that shape the perceptions, priorities, and decisions of female undergraduate students in the STEM disciplines. Based on the literature review, this study posits the following hypotheses for testing on the local population:

1. The array of environmental factors shaping female undergraduates’ study experiences in a Global South country is distinct from that observed in Global North ones.
2. There is no significant difference in the perception of environmental factors within the entire STEM between female students pursuing Engineering and those pursuing Natural Science degrees.

## Methodology

**Figure 2:**
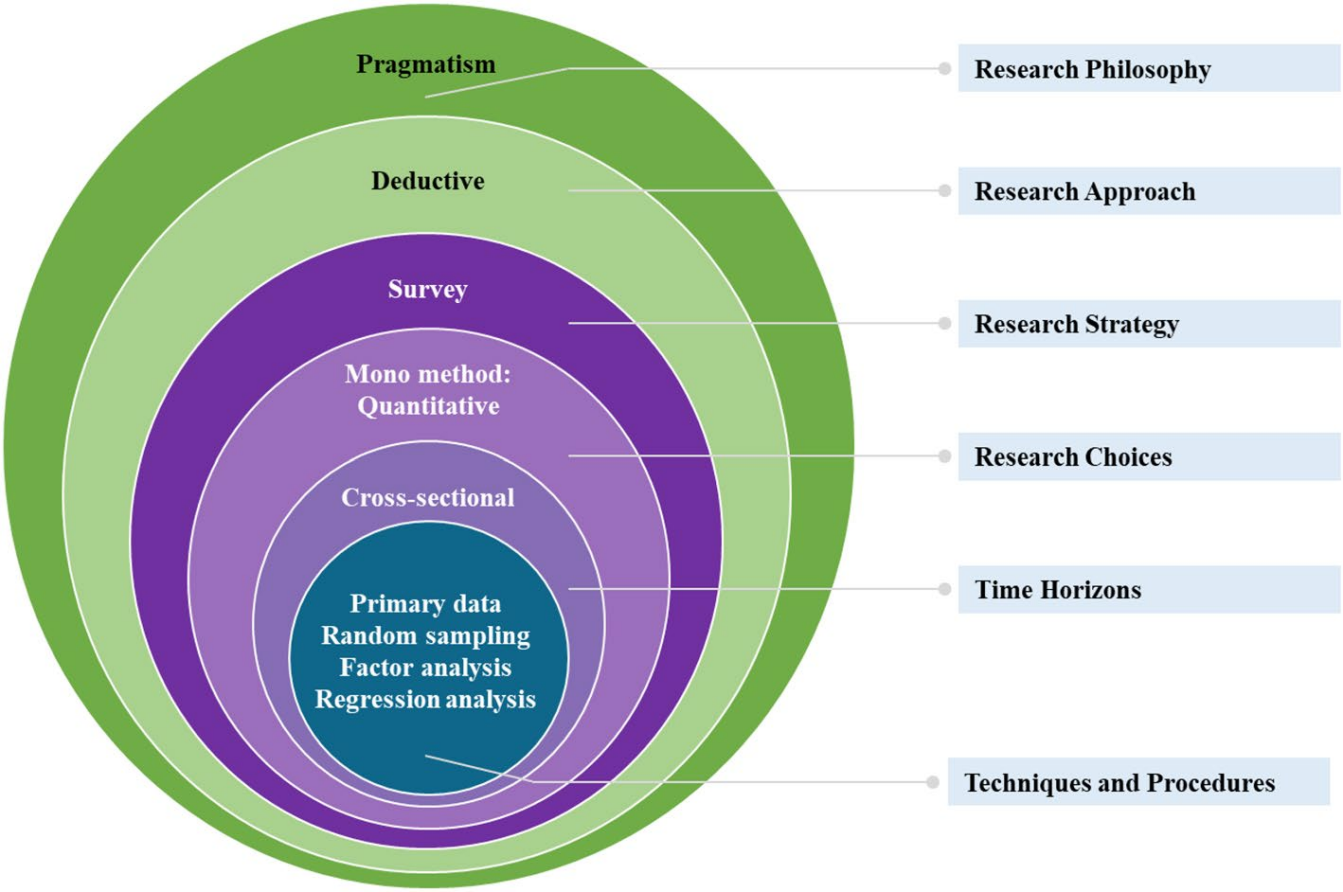
Saunders layered research framework.

The Saunders research onion was devised to offer a methodical methodology framework, which serves as a guiding structure for researchers as they navigate the intricate layers of the research process. This framework facilitates a systematic examination of research questions, beginning from the outermost layer and delving into the central core^40^. Figure 2 depicts the research onion designed for this study comprising six layers, namely, research philosophy, research approach, research strategy, research choices, time horizons, techniques and procedures.

The outermost layer deals with the research philosophy of pragmatism as its guiding principle. This study embraces a pragmatic approach, emphasizing the practical outcomes of its results. Its primary goal is to tackle real-world challenges associated with the underrepresentation of females in STEM fields, specifically engineering and IT.

In the next layer, a deductive research approach is chosen. The adoption of a deductive approach is grounded in the study's foundation upon Bandura's well-established social cognitive theory, from which empirically testable hypotheses are derived and rigorously examined within the specific contextual framework of Kazakhstan^41,42^. The Social Cognitive Theory framework chosen states that a person’s perception of learning is shaped by environmental factors^25^. These factors were described in the literature review section and established a foundation for constructing the survey, which represents the next layer of the onion and serves as a research strategy. Table 1 illustrates the survey questionnaire table, which was adapted from the one in an earlier study exploring students’ perceptions of why they choose engineering as a major^43^.

**Table 1:** Survey questionnaire based on the theoretical framework construct.

Research question	Theoretical construct	Survey question
How differently do female undergraduate students in STEM perceive the availability of learning facilities as a facilitator of their learning?	Presence of learning facilities	Availability of university facilities (i.e., IT labs, science labs, innovation hubs) encourages your interest in STEM subjects
		STEM development activities (specialized courses, competitions, industry-related collaborations) help you to build passion for learning
How differently do female engineering students in STEM perceive the presence of gender discriminative campus climate?	Gender discriminative campus climate	You have received a gender discriminative judgement from an instructor that discourages you to remain in your STEM pathway
		You feel that you need to "prove your worth" to be equally treated by male peers and your instructors
		Your instructors in your academic environment believe one needs to possess innate talent to succeed in STEM fields
How differently do female undergraduate students in STEM perceive the presence of supportive environment as a facilitator of their learning?	Presence of a supportive environment	The presence of at least one member of your family involved in STEM field motivates you to pursue a career in it as well
		The presence of a larger number of girls in your class encourages you to continue your STEM pathway
		Presence of a female faculty as a role model strengthens your feelings/affinity with the STEM fields
		The support you receive from female instructors helps you to maintain enthusiasm towards STEM major
How differently do female undergraduate students in STEM perceive the presence of gender-based societal stereotypes?	Wrong gender role expectation/stereotyping	You feel pressure due to societal stereotype, in which a woman is regarded as a "housewife" and "caretaker", but not a "career pursuer"
		People in your environment believe that the STEM related professions are more masculine and male-oriented ones
		Women working in STEM have fewer opportunities to achieve work-life balance
How differently do female undergraduate students in STEM perceive the employment prospects after graduation?	Clear vision of employment opportunities	You believe that there are sufficient employment options with respect to STEM-related positions
		You believe that male graduates are more likely to get career advancement in STEM positions
		You believe that male graduates are paid higher than women employee in STEM related jobs

The next level in the research onion is the research choice, focusing primarily on how data is collected in this study. A mono-method approach is adopted, given that the survey was crafted specifically to collect quantitative data using a Likert Scale questionnaire. The Likert Scale questionnaire was developed with a scale from 1 to 4, where 1 indicates "strongly agree” and 4 indicates "strongly disagree." The middle point of 3 was removed due to potential hidden biases that participants might not desire to expose, even though the survey is conducted fully anonymously^44^. The methodology of this study and the survey instrument were reviewed by the university’s Institutional Research Ethics Committee (IREC) and received prior approval (ID: 573/17052022). All methods were performed in accordance with relevant guidelines and regulations. Informed consent was diligently obtained from all participants prior to the commencement of the survey.

The time horizons layer is characterized as cross-sectional, denoting that the participant recruitment process spanned one academic year, commencing in September 2021 and concluding in April 2022. The research team opted for primary data collection, conducting the survey themselves and directly gathering responses from the participants, ensuring the integrity of the data collection process. The recruited participants for the study are female undergraduate students currently enrolled in STEM majors at an international English-medium institution located in Kazakhstan. The institution is the leading one in Central Asia, providing courses in the Western mode with a solid balance of male to female students’ ratio. That implies that the derived insights from this study and the overall situation on this issue might be even worse or more intense than in any other institution in the area.

The institution provides a broad spectrum of educational undergraduate and graduate programs where both STEM and non-STEM fields of study are offered. The participants of this study are students from two distinct schools, the School of Engineering, and the School of Sciences. Students in Chemical, Civil, Computer, Electrical, Mechanical and Robotics Engineering constitute the School of Engineering while students in the natural sciences of Biology, Chemistry, Mathematics and Physics constitute the School of Sciences.

A random sampling approach was employed as a part of the techniques and procedures layer, guaranteeing equal and unbiased opportunities for every member of the population to be chosen as a participant in the study. The total population of undergraduates pursuing one of the STEM degrees is 2525 (995 or 60% in Engineering and 1530 or 40% in the Sciences) with the percentage of female students standing at 27%. A total of 131 responses were collected from female students, an adequate sample for this population of 681 according to Cochran’s formula^45^. Of the 131 responses 52% came from the Sciences and 48% from Engineering, slightly skewing the participation in favour of the Sciences.

Out of the 131 participants, 34% were first-year, 27% were second-year, 23% were third year and 16% were fourth-year students. This distribution is slightly more skewed than the tapering seen in the actual population, as some students progressively change majors or even abandon their studies.

As a part of the techniques and procedures layer, this study employs a methodological approach centred on the quantitative analysis of survey data obtained from participants' responses. The research roadmap involves conducting an Exploratory Factor Analysis (EFA) to reduce the dimensionality and test the variables for multicollinearity. This instrument allows for the evaluation of whether items would correlate with the other factors, thereby reducing the number of factors. EFA is commonly performed to group variables based on their variances to discover the number of factors influencing certain variables^46^. Since the survey questions of this study have never been tested on the given population and factors have not been explored, the EFA was executed to examine the underlying structure of the variables. To validate the pattern of relationships between factors and their measuring items identified in the EFA construct, the confirmatory factor analysis (CFA) on the predetermined structure was performed. The CFA, in essence, confirms the construct during the EFA stage by verifying the explored factors representing each of the measuring items^47^.

Following the factor analysis, a logistic regression model that seeks to predict the critical factors characterizing the learning environment of female students pursuing degrees in both engineering and non-engineering fields was performed. The rationale for selecting an ordinal logistic regression model lies in the fact that it enables one to explain the relationship between the independent variables and the dependent variable in the form of a binary outcome^48^. This is particularly advantageous in identifying perceptual disparities between two groups of participants. The factor analysis and ordinal logistic regression analysis were both executed using Microsoft Excel and the statistical software tool IBM SPSS Statistics.

## Analysis

The survey was designed with five variables and fifteen items based on the theoretical framework. Cronbach’s alpha for this setup was computed at 0.81, a level sufficient to guarantee the internal reliability of the survey^49^. Table 2 below illustrates the summary descriptive statistics of the entire survey.

**Table 2:** Descriptive statistics of responses to each item (N = 131).

#	Question	Mean	Std. deviation	Skewness	Kurtosis
1	Availability of university facilities (i.e., IT labs, science labs, innovation hubs) encourage your interest in STEM subjects	3.11	0.79	− 0.75	0.36
2	STEM development activities (specialized courses, competitions, industry related collaborations) help you to build passion for learning	2.97	0.71	− 0.59	0.63
3	You have received a gender discriminative judgement from an instructor that discourages you to remain in your STEM pathway	1.93	0.77	0.52	− 0.1
4	You feel that you need to "prove your worth" to be equally treated by male peers and your instructors	2.71	0.92	− 0.27	− 0.78
5	Your instructors in your academic environment believe one needs to possess innate talent to succeed in STEM fields	2.23	0.71	0.42	0.2
6	The presence of at least one member of your family involved in STEM field motivates you to pursue a career in it as well	2.56	0.95	− 0.13	− 0.91
7	People in your environment believe that the STEM related professions are more masculine and male oriented	2.85	0.85	− 0.39	− 0.43
8	You believe that there are sufficient employment options concerning STEM-related positions	3.05	0.77	− 0.39	− 0.44
9	You believe that male graduates are more likely to get career advancement in STEM positions	2.80	0.82	− 0.3	− 0.43
10	You believe that male graduates are paid higher than women employees in STEM related jobs	2.84	0.88	− 0.42	− 0.54
11	The presence of a larger number of girls in your class encourages you to continue your STEM pathway	2.82	0.85	− 0.4	− 0.41
12	Presence of a female faculty as a role model strengthens your feelings/affinity with the STEM fields	3.17	0.83	− 0.71	− 0.23
13	The support you receive from female instructors helps you to maintain enthusiasm towards STEM major	2.93	0.8	− 0.42	− 0.24
14	You feel pressure due to societal stereotype, in which a woman is regarded as a "housewife" and "caretaker", but not a "career pursuer"	2.90	0.93	− 0.49	− 0.62
15	Women working in STEM have fewer opportunities to achieve work-life balance	2.94	0.87	− 0.52	− 0.38

The EFA was performed on the collected data based on the Likert scale survey results using principal component analysis (PCA) with the settings of the varimax rotation method and Kaiser normalization. The threshold value of an eigenvalue was set to be one or higher to validate only those factors whose variance was explained by rotated components^50^. The Kaiser–Meyer–Olkin measure of sampling adequacy was computed to be 0.705, indicating that the data sample is appropriate for conducting factor analysis^51^. An additional metric, Bartlett’s Test of Sphericity was significant (χ^2^ = 351.4; df = 28, p < 0.001), illustrating a strong relationship between variables and thus, the appropriateness of EFA^52^. Table 3 illustrates the pattern matrix, which summarizes the results of the PCA with derived loading values in it. In essence, the loading values show the correlations between the items and the factors. The survey items that appeared to have a higher correlation with the other components (factors) than the threshold value was eliminated to avoid a multicollinearity issue. Based on the components’ eigenvalues, the total number of factors was determined to be three. The survey questions were utilized to assign labels to the components, whereby factor 1 was denoted as *Career Stereotypes* (CS), factor 2 as *School Facilities* (SF), and factor 3 as *Female Instructor* (FI)".

**Table 3:** Pattern matrix with loading values between variables and components (factors).

Survey item	Item code	Component		
		Career stereotypes	School facilities	Female instructors
You believe that male graduates are more likely to get career advancement in STEM positions	CS2	0.870		
Women working in STEM have fewer opportunities to achieve work-life balance	CS4	0.771		
You believe that male graduates are paid higher than women employee in STEM related jobs	CS3	0.753		
People in your environment believe that the STEM related professions are more masculine and male-oriented ones	CS1	0.523		
STEM development activities (specialized courses, competitions, industry related collaborations) help you to build passion for learning	SF2		0.909	
Availability of university facilities (i.e.com labs, science labs, innovation hubs) encourage your interest in STEM subjects	SF1		0.830	
Presence of a female faculty as a role model strengthens your feelings/affinity with the STEM fields	FI1			0.854
The support you receive from female instructors helps you to maintain enthusiasm towards STEM major	FI2			0.819

**Figure 3:**
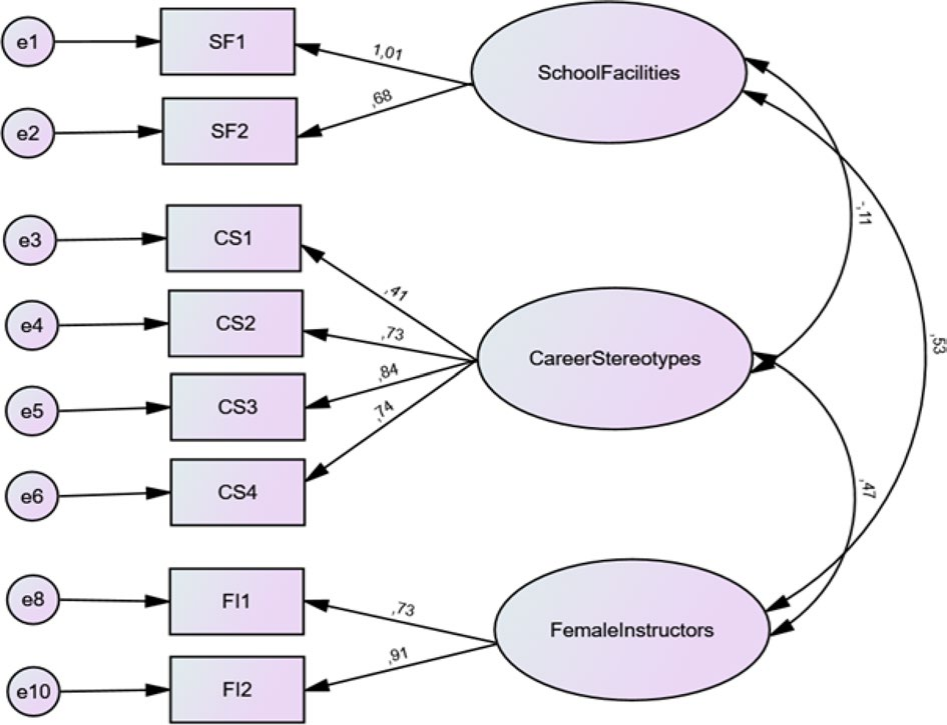
The structure of the variables and its measuring items.

The configuration and outcomes of the CFA model are presented in Fig. 3, where ovals represent factors, rectangles represent measuring variables, and circles represent error terms. A unidirectional arrow in the figure indicates a hypothesized pathway in which the variable at the tail of the arrow is posited to exert a causal influence on the variable at the head of the arrow, thereby serving as a visual representation of a causal relationship. Conversely, the double-headed arrows suggest the presence of a non-directional relationship between two factors.

The overall fit of the model falls within acceptable limits, indicating that the constructed model adequately validates the given construct. The coefficients above the arrows in Fig. 3, known as factor loadings, express the relationship between the given factor and the measuring variables in each construct. All standardized factor loadings are significant at p = 0.005. None of the factor loading values between the factor and measurable variables are below 0.4, as depicted in Fig. 3, and therefore all of them are considered in our model. While a coefficient closer to 1 suggests a higher positive correlation between the factor and variables, a practical rule of thumb focuses on factor loadings above 0.4 while disregarding the rest^53^. None of the factor loading values between the factor and measurable variables are below 0.4, as depicted in Fig. 3, and therefore all of them are considered in our model.

The *School Facility* 1 item holds the highest factor loading of 1.01 for school facilities, while *Career Stereotypes* 3 item holds the highest loading of 0.84 for career stereotypes, with *Career Stereotypes* 1 item holding the lowest loading of 0.41. For female instructors, *Female Instructors* 2 item holds the highest factor loading of 0.91, while *Female Instructor* 1 item also holds a significantly higher value of 0.73 compared to the 0.4 threshold. The arrows linking the three factors represent the covariances among them. The figure indicates a positive relationship of 0.53 between *School Facilities* and *Female Instructors*, a covariance value of 0.47 between *Career Stereotypes* and *Female Instructors*, and a slightly negative relationship of − 0.11 between *School Facilities* and *Career Stereotypes*.

## Results

The results of factor analysis provide a basis to reject the first hypothesis, because these three factors, namely, *Career Stereotypes, School Facilities*, and *Female Instructors* are aligned with the factors described in the literature. This implies that undergraduate students in the Global South country pursuing STEM disciplines encounter a similar array of environmental factors which influence their perception within STEM domains.

The descriptive statistics with a summary of the derived three factors in Table 4 illustrate that the *Female Instructor* factor has the highest total mean value (3,05 ± 0.73), while the *School Facilities* one has the lowest (2.86 ± 0.65) as applicable for School of Sciences. By interpreting this summary table in a more granular way, *Career Stereotypes* is recognized as the most discernible for the female students from School of Engineering as well as for the ones from School of Sciences, based solely on the mean values of the Likert scale survey results. The respective mean values are 3.02 ± 0.78 and 3.04 ± 0.68. The least recognizable factor for both respondents from both schools appears to be the School Facilities.

**Table 4:** Descriptive statistics of the established factors.

Factor	Faculty	n	Mean	Std. deviation	Skewness	Kurtosis
Career stereotypes	Engineering	54	3.02	0.78	− 0.86	0.5
	Sciences	77	3.05	0.62	− 0.49	0.43
	Total	131	3.04	0.68	− 0.74	0.77
School facilities	Engineering	54	2.90	0.68	− 0.57	0.03
	Sciences	77	2.83	0.63	− 0.23	0.06
	Total	131	2.86	0.65	− 0.38	0.07
Female instructors	Engineering	54	2.91	0.78	− 0.82	0.43
	Sciences	77	3.15	0.69	− 0.27	− 0.99
	Total	131	3.05	0.73	− 0.59	0.11

In order to assess the disparity in the perception of the identified factors among female students enrolled in the School of Engineering and School of Sciences, a binary logistic regression was employed. The model utilized for the analysis was based on the work conducted by^54^. The model allows to understand how one or more independent variables influence the likelihood of the dependent variable, which can take on values of either 1 or 0^48^. The value of 1 indicates responses of students from the School of Engineering, while 0 indicates responses of students from the School of Sciences. The independent variables considered in the model comprised of the three factors derived from the exploratory and confirmatory factor analyses. The Hosmer and Lemeshov test statistics were computed to be 3.13 (p > 0.05), indicating that the model adequately fits the data.

The results indicate that the variable *Female Instructors* yielded a statistically significant outcome at the 5% level of significance, whereas the variable *Career Stereotypes* yielded a significant result at the 10% level of significance (β = − 0.74, p < 0.05; β = 0.55, p < 0.1), respectively. The key output of this model is that there is a significant difference in perception of the *Female Instructors* factor by the female students from School of Engineering compared to those from School of Sciences. Female students from the School of Engineering were less likely to mention the *Female Instructors* factor as a facilitator towards pursuing their degree, as the regression coefficient (β) is negative (− 0.74). This contrasts with the female students who pursue pure science degrees from the School of Sciences.

**Figure 4:**
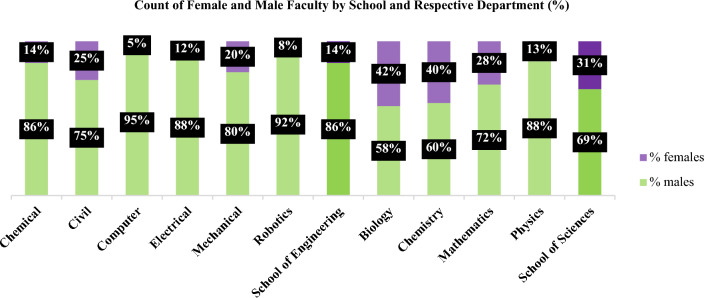
Gender distribution of faculty at the institution of both Schools and Departments.

According to the responses, the assumption that female mentorship benefits female students pursuing their majors was supported. However, the recognition of this factor seemed to differ between female students from the two schools, which contradicts the second hypothesis proposed. One key difference in this phenomenon is that the number of female mentors and role models in the science majors is significantly higher compared to the other majors. This suggests that the impact of female faculty and instructors on students in Engineering disciplines may not be significant, potentially due to insufficient representation. Figure 4 can provide further insight into this explanation.

Figure 4 demonstrates that the male faculty members at the School of Engineering Departments significantly outnumber their female counterparts. (14% female vs 86% male).

For example, 14% female against 86% male in Chemical Engineering, 25% female against 75% male in Civil Engineering, 5% female against 95% male in Computer Engineering, 12% female against 88% male in Electrical Engineering, 20% female against 80% male in Mechanical Engineering, 8% female against 92% male in Robotics Engineering. Particularly in the Civil Engineering, the percentage of female faculty reaches 25% of the entire Department, which is the highest rate compared to the other branches. In some Departments, this situation is even worse, particularly in the Computer Engineering and Robotics Departments.

The School of Sciences portrays a more diversified environment in each Department, considering the relatively elevated percentage of female academics, accounting for 31%. Despite the presence of relatively high percentages of female faculty members in the Biology and Chemistry Departments of the school, amounting to 42% and 40% respectively, a gender parity scenario is yet to be achieved. In contrast, the Mathematics and Physics Departments continue to exhibit a notable underrepresentation of female academics.

Female students from the School of Engineering were found to have significantly different perceptions of the *Career Stereotypes* factor compared to their counterparts from natural sciences. Female students studying engineering disciplines are at a higher risk of being affected by gender stereotypes that associate male dominance with their future work environments compared to those studying natural sciences, as the regression coefficient (β) of the model is positive (0.55). It appears that female engineering students acknowledge the potential for discrimination in their future careers and perceive that the workplace conditions are unequal and biased towards male colleagues, unlike female students in non-engineering fields. Thus, it refutes the second hypothesis, as the feelings and expectations regarding future careers among female students in both categories are not similar.

All the participants in the study exhibited a similar pattern regarding the School Facilities factor. Female students from both schools treated this factor in an equal manner, which lends support to the second hypothesis. These findings suggest that the provision of university facilities and STEM development activities may enhance learning experiences for female undergraduates in STEM majors, irrespective of any subgroup.

One of the main limitations of this study was the limited sample size, which was confined to a single university. Increasing the sample size could provide a broader and more representative perspective of the current study, which may be considered as suggestion for future research. A further limitation of this study is that some of the factors and questions only assessed external factors. Additionally, future studies could benefit from including additional factors that measure the affinity or internal emotions of female students. Lastly, as this research examined the perceptions of female undergraduate students and not their intention to continue pursuing a STEM path, it is suggested to include a binary variable to measure their future intentions to either remain in or exit the STEM pipeline.

## Discussion

In order to investigate if the environmental factors found in international literature can account for the gender imbalance in career choices between engineering and natural sciences among female undergraduate students in Kazakhstan, comprehensive survey among female STEM majors enrolled in the top university in Kazakhstan was conducted. The value of Cronbach’s alpha to guarantee the internal reliability of the survey, Kaiser–Meyer–Olkin to measure the sampling adequacy and Bartlett’s Test of Sphericity for measuring strong correlation between variables was calculated and found to be significant. A model was developed using logistic regression to predict the key factors that define the learning environments of female students pursuing degrees in both engineering and non-engineering fields, following a factor analysis. The following observations merit discussion.

- The results reveal that the common environmental factors impacting female students worldwide also exist in this country in the Global South. This study identifies career stereotypes, female instructors, and school facilities as the prevalent factors in Kazakhstan. Furthermore, the covariance value suggests that school facilities have a positive association with female instructors, as well as career stereotypes and female instructors.
- A binary logistic regression analysis was performed to assess the perception disparity among female students. In contrast to previous literature, our study found that Engineering students were less likely to perceive female instructors as supportive due to the lack of visible female role models. On the other hand, they were more likely to identify career stereotypes as a hindrance compared to female students from the School of Sciences.
- As anticipated, female engineering students displayed a strong awareness of the career stereotypes that they are likely to encounter in their future workplaces. This heightened perception may lead to a lower rate of young female engineering graduates entering male-dominated fields as they anticipate discrimination, despite the increasing number of women enrolling in STEM programs.
- Based on logistic regression, no significant difference was perceived in terms of school facilities by both schools. However, School of Engineering students perceived career stereotypes and experiences with female instructors significantly differently than the school of sciences which act as a major deterrent for female students pursuing STEM majors and careers and therefore rejecting our second hypothesis.
- These findings contribute to the ongoing discourse surrounding the "leaky pipeline" and “glass ceiling” phenomena in STEM which make it difficult for women to balance career advancement with lack of support, recognition, and an overwhelming workload, especially in Kazakhstan. They shed light on the intricate factors that underline the attrition of women within the engineering, manufacturing and IT fields, providing valuable insights for policymakers, educators, and stakeholders. As we continue to grapple with the challenges of diversity and inclusion in STEM, this research underscores the urgent need for targeted interventions and systemic changes to ensure that the pipeline remains secure at various stages for female participants.

## Conclusion

Environmental factors can significantly influence the experience of female undergraduates in universities, and there are indeed distinctions in these factors between Global South (developing) and Global North (developed) countries. In the Global South, socio-cultural norms might be more conservative, limiting women's participation in certain fields, especially against economic constraints, safety concerns, and infrastructural challenges. In contrast, while women in Global North countries have greater institutional support and a diverse student body, they still face challenges in male-dominated fields like STEM, such as persisting gender stereotypes, and issues of work-life balance.

This study demonstrates, however, that when the focus is narrowed on factors directly related to the learning environment (presence of learning facilities; gender discriminative campus climate; the presence of a supportive environment; wrong gender role expectation/stereotyping; and clear vision of employment opportunities) there is no discernible difference between the observations from Kazakhstan and those delineated in the literature from a host of Global North country studies.

It is essential to note that generalizing this outcome can be limited by the wide range of diversity within both Global South and Global North countries. Experiences can vary widely within each region due to individual countries and institutional contexts. It is essential to broaden the scope of the study to include a wider range of Global South countries in order to reach conclusions with wider applicability.

The second, and somewhat surprising, outcome of this study is that there are significant differences in the perception of environmental factors between female students pursuing Engineering and those pursuing Natural Science degrees. Female students in Engineering often grapple with challenges like gender stereotyping in a traditionally male-dominated field, a lack of female role models, and cultural biases against women in engineering roles. In contrast, those in Natural Sciences, such as biology or chemistry, might encounter a more balanced gender representation but still face specific stereotypes about "suitable" roles within the field. However, both fields can share concerns about analytical ability perceptions, safety during practical work, and work-life balance challenges. Individual experiences, though, vary widely based on institution, culture, and personal background.

In light of the insights gleaned from this study, academic administrations can harness the knowledge presented here to refine their strategies for fostering gender inclusivity within STEM disciplines. By recognizing the shared challenges faced by female students in both engineering and natural sciences, institutions can tailor support mechanisms, mentorship programs, and awareness initiatives to address field-specific obstacles^55^. The vital role that administrators can play in addressing diversity, equity, and inclusion issues in academic institutions is underscored, and the importance of proactive efforts to create an inclusive learning environment transcends the geographical distinctions and challenges identified in the study^56^.

## Data Availability

The datasets generated during and/or analysed during the current study are available from the corresponding author on reasonable request.

## References

[1] The United Nations Educational, Scientific and Cultural Organization (UNESCO). *Global Education Monitoring Report–Gender Report: A new generation: 25 years of efforts for gender equality in education.* https://gem-report-2020.unesco.org/gender-report/progress-towards-gender-parity-in-education-is-undeniable/ (2020).

[2] Chavatzia, T. *Cracking the code: girls’ and women’s education in science, technology, engineering and mathematics (STEM).* https://unesdoc.unesco.org/ark:/48223/pf0000253479 (2017).

[3] Dados N, Connell R (2012). The global south. Contexts.

[4] Dzhuraev S (2021). How southern is Central Asia?. APSA-CP Newsl. (American Political Science Association).

[5] Akhmadi S, Tsakalerou M (2023). Exploring gender imbalances in innovation and entrepreneurship: Evidence from a global south country. IJGE.

[6] World Bank Group. *A New Growth Model for Building a Secure Middle Class*. http://hdl.handle.net/10986/29792 (2018).

[7] Tsakalerou M, Perveen A, Ayapbergenov A, Rysbekova A, Bakytzhanuly A (2022). Understanding the factors influencing women’s career trajectories in STEM education in Kazakhstan. Int. Conf. Gender Res..

[8] Ho M-T (2020). An analytical view on STEM education and outcomes: Examples of the social gap and gender disparity in Vietnam. Child Youth Serv. Rev..

[9] Tsakalerou, M. & Akhmadi, S. Women and innovation: The missing link. In *5th European International Conference on Industrial Engineering and Operations Management* 1720–1729 (2022).

[10] United Nations Entity for Gender Equality and the Empowerment of Women (UN Women). *UN Women Kazakhstan One-Pager.* https://eca.unwomen.org/en/digital-library/publications/2022/10/un-women-kazakhstan-one-pager (2022).

[11] StatGov.KZ. *Statistics of Education, Science, and Innovation.* https://stat.gov.kz/ru/industries/social-statistics/stat-edu-science-inno/ (2022).

[12] Dmitrienko AS, Kuzhabekova AS (2023). Employment of STEM graduates in Kazakhstan: Current situation. Bull. Turan Univ..

[13] Clark Blickenstaff J (2005). Women and science careers: Leaky pipeline or gender filter?. Gend. Educ..

[14] Resmini M (2016). The ‘Leaky Pipeline’. Chem. Eur. J..

[15] Shapiro M (2015). Middle school girls and the “Leaky Pipeline” to leadership: An examination of how socialized gendered roles influences the college and career aspirations of girls is shared as well as the role of middle level professionals in disrupting the influence of social gendered messages and stigmas. Middle Sch. J..

[16] Almukhambetova A, Torrano DH, Nam A (2023). Fixing the leaky pipeline for talented women in STEM. Int. J. Sci. Math. Educ..

[17] Syzdykova Z (2017). Key aspects of the Kazakh religious identity. Eur. J. Sci. Theol..

[18] The U.S. Agency for International Development (USAID). *Making Their Way in Male-Dominated Professions*. https://www.usaid.gov/kazakhstan/news/making-their-way-male-dominated-professions (2021).

[19] Glass Ceiling Commission. *A Solid Investment : Making Full Use of the Nation’s Human Capital*. https://ecommons.cornell.edu/items/23c55395-2df6-4490-8234-b72b46895564 (1995).

[20] Shtey D (2022). Gender in Kazakhstan’s Mining Industry.

[21] Zhailaubayeva A (2021). The Effect of Organizational Culture to Female Career in Kazakhstan.

[22] Kuzhabekova A, Janenova S, Almukhambetova A (2018). Analyzing the experiences of female leaders in civil service in Kazakhstan: Trapped between economic pressure to earn and traditional family role expectations. Int. J. Public Adm..

[23] Nguyen LT, Taylor G, Gibson P, Gordon R (2023). Advancing a critical social psychological perspective on women’s leadership: A case illustration from the Global South. Appl. Psychol..

[24] Vargas-Solar G (2022). Intersectional study of the gender gap in STEM through the identification of missing datasets about women: A multisided problem. Appl. Sci..

[25] Bandura A (1986). Social Foundations of Thought and Action: A Social Cognitive Theory.

[26] Jensen LE, Deemer ED (2019). Identity, campus climate, and burnout among undergraduate women in STEM fields. Career Dev. Q..

[27] Victorino C, Denson N, Ing M, Nylund-Gibson K (2022). Comparing STEM majors by examining the relationship between student perceptions of campus climate and classroom engagement. J. Hispanic Higher Educ..

[28] Maltese AV, Cooper CS (2017). STEM pathways: Do men and women differ in why they enter and exit?. AERA Open.

[29] Chachashvili-Bolotin S, Milner-Bolotin M, Lissitsa S (2016). Examination of factors predicting secondary students’ interest in tertiary STEM education. Int. J. Sci. Educ..

[30] Almukhambetova A, Kuzhabekova A (2020). Factors affecting the decision of female students to enrol in undergraduate science, technology, engineering and mathematics majors in Kazakhstan. Int. J. Sci. Educ..

[31] Martin-Hansen L (2018). Examining ways to meaningfully support students in STEM. IJ STEM Ed.

[32] Tandrayen-Ragoobur V, Gokulsing D (2022). Gender gap in STEM education and career choices: What matters?. JARHE.

[33] Shin JEL, Levy SR, London B (2016). Effects of role model exposure on STEM and non-STEM student engagement: Role model. J. Appl. Soc. Psychol..

[34] Almukhambetova A, Kuzhabekova A (2021). Negotiating conflicting discourses. Female students’ experiences in STEM majors in an international university in Central Asia. Int. J. Sci. Educ..

[35] Piatek-Jimenez K, Cribbs J, Gill N (2018). College students’ perceptions of gender stereotypes: Making connections to the underrepresentation of women in STEM fields. Int. J. Sci. Educ..

[36] Antoshchuk I (2021). Moving through the STEM pipeline: A systematic literature review of the gender inequality in Russian engineering. Monit. Public Opin. Econ. Soc. Changes..

[37] Westoby C, Dyson J, Cowdell F, Buescher T (2021). What are the barriers and facilitators to success for female academics in UK HEIs? A narrative review. Gender Educ..

[38] Syzdykbayeva, R. *Exploring Gender Equality in STEM Education and Careers in Kazakhstan*. 189–225 https://unesdoc.unesco.org/ark:/48223/pf0000375106 (2020).

[39] Khitarishvili T (2016). Gender dimensions of inequality in the countries of Central Asia, South Caucasus, and Western CIS. SSRN J..

[40] Melnikovas A (2018). Towards an explicit research methodology: Adapting research onion model for futures studies. J. Futures Stud..

[41] Schunk DH, DiBenedetto MK (2020). Motivation and social cognitive theory. Contemp. Educ. Psychol..

[42] Iovino F, Tsitsianis N (2020). The methodology of the research. Changes in European Energy Markets.

[43] Young E (2014). Understanding Women’s Choices to Enroll in Engineering: A Case Study.

[44] Chyung SYY, Roberts K, Swanson I, Hankinson A (2017). Evidence-based survey design: The use of a midpoint on the likert scale. Perf. Improv..

[45] Noordzij M (2010). Sample size calculations: Basic principles and common pitfalls. Nephrol. Dial. Transplant..

[46] Yong AG, Pearce S (2013). A beginner’s guide to factor analysis: Focusing on exploratory factor analysis. TQMP.

[47] Miksza P, Elpus K (2018). Design and Analysis for Quantitative Research in Music Education.

[48] Stoltzfus JC (2011). Logistic regression: A brief primer. Acad. Emerg. Med..

[49] Bland JM, Altman DG (1997). Statistics notes: Cronbach’s alpha. BMJ.

[50] Gunuc S, Kuzu A (2015). Student engagement scale: Development, reliability and validity. Assess. Eval. High. Educ..

[51] Adams A-M (2021). Characteristics of the preschool home literacy environment which predict writing skills at school. Read. Writ..

[52] Chouhan SS, Kathuria A, Sekhar CR (2021). Examining risky riding behavior in India using motorcycle rider behavior questionnaire. Accid. Anal. Prev..

[53] Natalya L, Purwanto CV (2018). Exploratory and confirmatory factor analysis of the Academic Motivation Scale (AMS)–Bahasa Indonesia. Makara Hubs-Asia.

[54] Liu X, Koirala H (2012). Ordinal regression analysis: Using generalized ordinal logistic regression models to estimate educational data. J. Mod. Appl. Stat. Methods.

[55] Wolf E, Brenning S (2023). Unlocking the power of mentoring: A comprehensive guide to evaluating the impact of STEM mentorship programs for women. Soc. Sci..

[56] Taylor JE (2023). The role of research leaders in enhancing diversity, equity, and inclusion: Directions from current research and opportunities for systemic organizational transformation. J. Res. Adm..

